# Potential health risk assessment of toxic metals contamination in clay eaten as pica (geophagia) among pregnant women of Ho in the Volta Region of Ghana

**DOI:** 10.1186/s12884-020-02857-4

**Published:** 2020-03-14

**Authors:** Nii Korley Kortei, Alice Koryo-Dabrah, Papa Toah Akonor, Nana Yaw Barimah Manaphraim, Matilda Ayim-Akonor, Nathaniel Owusu Boadi, Edward Ken Essuman, Clement Tettey

**Affiliations:** 1 Department of Nutrition and Dietetics, School of Allied Health Sciences, University of Health and Allied Sciences, PMB 31, Ho, Ghana; 2 Department of Food Processing and Engineering, CSIR- Food Research Institute, P.O. Box M20, Accra, Ghana; 3 Department of Nutrition and Food Science, School of Biological Sciences, College of Basic and Applied Sciences, University of Ghana, P.O. Box LG 25, Accra, Ghana; 4 Department of Medical Laboratory Sciences, School of Allied Health Sciences, University of Health and Allied Sciences, PMB 31, Ho, Ghana; 5 Animal Health and Food Safety Division, CSIR- Animal Research Institute, P.O. Box AH20, Achimota, Ghana; 6 Department of Chemistry, Kwame Nkrumah University of Science and Technology, Kumasi, Ghana; 7 Department of Biomedical Sciences, School of Basic and Biomedical Sciences, University of Health and Allied Sciences, PMB 31, Ho, Ghana

**Keywords:** Pica, Geophagy, Clay, Volta region, Pregnant women, Risk assessment, Ghana

## Abstract

**Introduction:**

Geophagia although pleasurable and somewhat a necessity among pregnant women, also comes along with its own attendant problems such as exposure to potentially hazardous substances like bacteria, fungi, helminthes and ova, radioactive materials, and toxic elemental minerals in the soil depending on the geographical location.

**Methodology:**

This study evaluated the potential health risk involved during the exposure of pregnant women to toxic elemental minerals via the consumption of clay as pica (geophagia). Elemental mineral analysis was carried out using Buck Scientific 210VGP Flame Atomic Absorption Spectrophotometer (Buck Scientific, Inc. East Norwalk, USA). Risk assessment methods were also used to ascertain the various risks factors and the overall risk level.

**Results:**

Concentrations of the macro elements investigated were 1.38 ± 1.5, 2.40 ± 1.5, 7.74 ± 1.5, 4.01 ± 1.0, 13.24 ± 2.2 and 13.76 ± 2.1 mg/Kg for iron (Fe), copper (Cu), zinc (Zn), potassium (K), magnesium (Mg) and sodium (Na) respectively. While that for the micro elements were 1.63 ± 0.03 μg/Kg, 4.72 ± 0.8, 0.53 ± 0.02 and 1.85 ± 0.3 mg/kg respectively for arsenic (As), manganese (Mn), lead (Pb) and nickel (Ni). Estimated Daily Intake (EDI), Hazard Quotient (HQ), Target Hazard Quotient (THQ) and Total Target Hazard Quotient (TTHQ) values ranged 0.611–5.44 (mg/kg Bw/day), 6.26 × 10^− 4^ – 106.5, 0.067–10.34 and 15 respectively.

**Conclusion:**

There is the likelihood of posing adverse health problems when clay samples obtained from Anfoega which is sited in the Volta region of Ghana is consumed due to the fact that the HQ’s of these elemental minerals were > 1 which points to high content of Manganese (Mn) and Nickel (Ni). It is also likely to cause adverse health problems in an individual’s life time since THQ for Arsenic, Lead and Nickel were above 1. Ultimately, the cumulative effect of these toxicants were exceedingly great (≤ 15) which implied a high level of unsafety associated with this clay. Per the results from this study, it is not safe for pregnant women to consume clay as pica since these toxic elements may cause detrimental effects on the foetus of the unborn child.

## Background

Pica is universally described as the obstinate ingesting of substances that provide no nutrients and is aptly categorized as a syndrome associated with eating [1, 2]. WHO [3] highlighted that this captures a wide range of excessive and insistent consumption of both nutritional and non-nutritional objects without the intention of deriving nutritional benefits but rather some satisfaction or pleasure. Accordingly, pica may include eating of dust, earth, soil or clay (geophagia), corn or laundry starch (amylophagia) likewise ice or freezer frost (pagophagia), and a host of other materials as elaborated by [2].

Geissler et al. [4] reiterated that geophagy or geophagia is utterly applicable to the eating of soil which is most common. Its practiced daily amongst healthy school going children in Kenya: it is not stigmatized and continues well into their period of adolescence. Geophagy is mostly practiced by adult pregnant women and sometimes young women. By implication, geophagy is probably much more prevalent in the tropics than previously estimated. Archival records shown by [5] suggests that women of the Ewe tribe in Ghana, for instance, can consume an average quantities of 30 g of clay per day. In Uganda, according to reports by Kilbride and Kilbride [6] as well as Ziegler [7] also suggest that women have a penchant for the peculiar taste and flavor of clay obtained from mounds of termite, ant hills or walls of the hut, of which they will often cook over the fire for its improvement before consumed. An inadvertent and common form of geophagy occurs when individuals enjoy inhaling dust arising from the soil prior to rainfall or drizzle [5].

Clay consumption is a common practice especially among women and is often linked with pregnancy, famine, idleness and insufficiency [8, 9]. In Ghana, it is estimated that about 28% of women of reproductive age who practice geophagy, consume a daily average of 70 g of clay [10]. Apparently, eating of clay and or soil as a habit was observed to be less frequent in boys than in girls. Furthermore, soil eating in boys is reported to decrease with age [11]. Geophagia has been shown to be prevalent among females in the teenage bracket and that by intensifying the education of these females, geophagia and its consequences can be minimized significantly [12].

Harmful heavy metals such as arsenic, lead, mercury and cadmium have been confirmed to have a strong correlation with human health according to some research works [9, 13]. In the same light, other researchers linked geophagy to potential bacterial, fungal and parasitic infections [14, 15] while there could also be a potential exposure to radioisotopes [16] in the soil via geophagia. Notwithstanding, some beneficial microbiological health effects associated with geophagia in humans include the use of kaolin (a form of clay) to treat diarrhoea and improve upon bioactivities [11]. A similar phenomenom was demonstrated by Ngole et al. [17] in their study of the physicochemical properties of geophagic clayey soils from South Africa and Swaziland. The presence of iron oxide in clay and the water retention capacity of clay was also found to alleviate anemia and diarrhea respectively.

Geophagia has taken another twist in Ghana where there has been reports of craving for geophagical soils which are commercially mined or excavated from known (and usually uncontaminated) sources at depth, rather than from the surface. Likewise, a penchant to eat termite mounds owing to its peculiar aroma and other attributes as pica by pregnant women.

Although clay or soil consumption in Ghana has gone on for a very long time, perusal of pertinent literature reveals scanty publications on the levels of heavy metals and the risks assessments likewise how it impacts on consumers and its health implication to affect policy formulation. Some studies done by previous researchers such as Macheka et al., 2016 [18], Meel, 2012 [19], Mensah et al., [2] and Nkansah et al., [20] have pointed to varied health problems associated with its consumption. The objective of this study was therefore to assess the potential health risk associated with clay prepared for consumption as pica from Anfoega by pregnant women in the Volta Region of Ghana as well as people from immediate neighboring countries Togo, Benin, Burkina Faso etc. who may consume it.

## Methods

### Study area, location

The Kaolin samples were purchased from Anfoega in the Volta Region. Anfoega is positioned in North Dayi district of the Kpando Municipality of the Volta Region, Ghana. It has geographical coordinates of 6° 53′ 0“ North, 0° 18’ 0” East and its original name (with diacritics) is Anfoega Akukome.

### Sampling of kaolin

Anfoega in the Volta region of Ghana was where baked kaolin samples were obtained from. It’s a location where majority of the kaolin ore is mined and processed (moulded and baked) for distribution and sale to markets in and around Ghana (especially Togo and Benin) for consumption. They were packaged in plastic containers and kept in low freezing temperatures (ice chest) at 4 °C and transported under aseptic conditions to the laboratory.

### Sample preparation for mineral analysis

The clay ore specimens were ground with mortar and pestle into powder and sieved using a 0.1 mm mesh. About 100 ml of distilled water was added to 100 g of the weighed specimen. The specimen was then placed on a shaker at 125 rpm for 12 h and then allowed to settle. The samples were filtered using a whatman 40 filter paper. The supernatant was then used for the analysis.

### Determination of mineral elements in clay

The dry ashing method was used for atomic absorption spectrophotometer (AAS) analysis as described by AOAC [21]. One percent (1%) nitric acid was used to wash all glass wares followed by demineralised water. Three millilitres (3 ml) each of the clay supernatants were weighed into platinum crucibles. The crucible and the test portion were placed in a Muffle furnace at a temperature of 550 °C for 8 h. The crucible with ash was put in a desiccator to cool. Five millilitres (5 ml) of nitric acid of mass fraction not less than 65%, having a density of approximately ρ (HNO_3_) = 1400 mgml^− 1^ was added, ensuring that all the ash came in contact with the acid and the resultant solution heated on hot plate until the ash was dissolved. Ten millilitres (10 ml) of 0.1 mol l^− 1^ nitric acid was added and filtered into 50 ml volumetric flask. The resultant solution was topped up to the mark with 0.1 mol l^− 1^ nitric acid. Blank solution was treated the same way as the sample. Absorbance values at appropriate wavelengths of the interested metal in the sample solution were read using the Buck Scientific 210VGP Flame AAS (Buck Scientific, Inc. East Norwalk, USA). Cathode lamps used were copper (Cu) (wavelength 324.8 nm, lamp current 1.5 mA), iron (Fe) (wavelength 248.3 nm, lamp current 7.0 mA), manganese (Mn) (wavelength 279.5 nm, lamp current 3.0 mA), lead (Pb) (wavelength 217.0 nm, lamp current 3.0 mA) and zinc (Zn) (wavelength 213.9 nm, lamp current 2.0 mA). Air/acetylene gas was used for all the analyses. Calibration curves made up of a minimum of three standards were used to detect the metal contents of the samples.

### Human health risk assessment of heavy metals in clay

In the risk assessment involved with the consumption of contaminated clay samples pose to humans, several health risk estimation methods have been proposed and used by some researchers [22–24]. One method is the Estimated Daily Intake (EDI), which helps to identify the quantity of pollutant consumed daily [25]. The EDI of potentially toxic elements (PTE) relies on the concentrations of PTE in the clay and the daily clay consumption. In addition, human body weight has an important influence on the tolerance to contaminants [25].

### Tolerable daily intake and estimated daily intake

The estimated daily intake (EDI) depends on the metal concentration, food consumption, and body weight. Table 3 shows the Permitted Daily Intake (PMTDI) of the heavy metals. To evaluate the risk of heavy metals from clay consumption at the extreme, we made the following assumptions in this research: the ingested dose was equal to the absorbed pollutant dose [20]; cooking (baking) has no effect on the pollutants [26]; the average adult body weight of Ghanaians was 60 kg [27]; Average daily consumption of clay in Ghana is 70 g clay per day [10]. Therefore, the EDI of heavy metals for adults was calculated as follows:

1 $$ EDI=\frac{C\;x\;C\; cons}{Bw} $$

where C is the concentration of heavy metals in clay sample (mg/kg wet weight), C cons is the average daily consumption of clay in the local area (70 g/day Bw) [10], and Bw represents the female body weight (60 kg) [27]. Table 1 shows the international guidelines and exposure parameters used for the risk estimations.

**Table 1:** Exposure parameters used for the health risk estimations via consumption of clay [27] (US EPA)

Parameter	Unit	Child	Adult
Body Weight (BW)	Kg	15	75
Exposure	Days/ years	365	365
Frequency (EF)			
Exposure	Years	6	30
Duration			
Ingestion Rate (IR_clay_)	mg/day	200	100
Average Time (AT)	Days/years		
For carcinogenic		365 × 70	366 × 70
For non-carcinogenic		365x ED	365x ED

### Determination of target Hazard quotient (THQ)

Target hazard quotients (THQ) were developed by the United States Environmental Protection Agency for the estimation of potential health risks associated with long term exposure to chemical pollutants. THQ is a ratio between the measured concentration and the oral reference dose, weighed by the length and frequency of exposure, amount ingested and body weight. THQ value is a dimensionless index of risk associated with long term exposure, amount ingested and body weight.

The THQ, the ratio of the exposure dose to the reference dose (RfD), represents the risk of non-carcinogenic effects. If it is less than 1, exposure level is less than the RfD. This indicates the daily exposure at this level is unlikely to cause adverse effects during a person’s lifetime, and vice versa.

The dose calculations were performed using standard assumptions from the integrated USEPA risk analysis [26]. The model for estimating THQ was determined by the following equation [26]:

2 $$ THQ=\frac{EFr\ x\  EDtot\ x\  FIR\ x\ C}{RfDo\ x\  Bw\ x\  ATn}x\;{10}^{-3} $$

where EFr is the exposure frequency (350 days/year); EDtot is the exposure duration (30 years); FIR is the clay ingestion rate (g/day), and 10^− 3^ is the unit conversion factor; C is the heavy metal concentration in clay (mg/kg wet weight); RfDo is the oral RfD (mg/kg-day); Bw is the average adult body weight (60 kg); and ATn is the average exposure time for non-carcinogens (365 days/year × number of exposure years, assuming 30 years).

### Non-carcinogenic effect

3 $$ HQ=\frac{EDI}{RfD} $$

where HQ is the hazard quotient and RfD is the reference dose (mg kg^− 1^ day^− 1^). HQ values of < 1 signify unlikely adverse health effects, while HQ values > 1 indicate a likely adverse health effect.

### Carcinogenic risk assessment

Carcinogenic risk assessment estimates the probability of an individual developing cancer over a lifetime due to exposure to the potential carcinogen is presented in Table 1.

In this assessment, a caner slope factor was used to convert the EDI of the heavy metals over a lifetime exposure to risk of an individual developing cancer [28].

$$ \mathrm{Risk}=\sum \mathrm{n},\mathrm{I}=1\kern0.5em \mathrm{EDI}\ \mathrm{x}\ \mathrm{CSF} $$

$$ \mathrm{CSF}=\mathrm{Cancer}\ \mathrm{Slope}\ \mathrm{Factor} $$

### Total target Hazard quotient

In this study, the total THQ was expressed as the arithmetic sum of the individual metal THQ values according to the method of [26]:

4 $$ Total\  THQ\ (TTHQ)= THQ\ \left( toxicant\ 1\right)+ THQ\ \left( toxicant\ 2\right)+ THQ\ \left( toxicant\ n\right) $$

### Statistical analysis

Results obtained was analyzed using IBM SPSS statistics version 22.0. Elemental minerals concentrations were obtained from duplicates and presented as mean concentration and standard deviations.

## Results

Results of the different concentrations of minerals both micro and macro elements are presented in Tables 2 and 3. Concentrations of the macro elements investigated were 1.38 ± 1.5, 2.40 ± 1.5, 7.74 ± 1.5, 4.01 ± 1.0, 13.24 ± 2.2 and 13.76 ± 2.1 mg/Kg for Fe, Cu, Zn, K, Mg and Na respectively. While that for the micro elements were 1.63 ± 0.03 μg/Kg, 4.72 ± 0.8, 0.53 ± 0.02 and 1.85 ± 0.3 mg/kg respectively for As, Mn, Pb and Ni.

**Table 2:** Concentrations of essential macro and trace elements in clay samples

Mineral Element	Concentration (mg/Kg) (Mean ± SD)	Recommended Dietary Intake (RDI), WHO [28]
Iron	1.38 ± 1.5	18 mg
Copper	2.40 ± 1.5	0.9 mg
Zinc	7.74 ± 1.5	11 mg
Potassium	4.01 ± 1.0	3100–3500 mg
Magnesium	13.24 ± 2.2	280–350 mg
Sodium	13.76 ± 2.1	500–2400 mg

**Table 3:** Mean concentrations of toxic trace elements in clay sample

Mineral element	Mean Concentration (Mean ± SD)	WHO/FAO PMTDI (μg/Kg BW/day)	PMTDI for 60
Arsenic	1.63 ± 0.03 μg/Kg	3.0	180
Manganese	4.72 ± 0.8 mg/Kg	4.9 mg/Kg	294
Lead	0.53 ± 0.02 mg/Kg	3.0	180
Nickel	1.85 ± 0.3 mg/Kg	5.0	300

### Reference doses for the various elemental minerals were of range 3.0 × 10^− 4^ – 0.14 mg kg^− 1^ day^− 1^ as used in this study (Table 4)

Estimated Daily Intakes of toxic metals in clay samples of this study recorded range values of 0.611–5.44 (mg/kg Bw/day).

**Table 4:** Reference doses (RFD) mg kg^− 1^ day^− 1^ for heavy metals used in this study

Heavy metals	Reference Doses	Cancer slope factor	References
Arsenic	3.0 × 10^−4^	1.50	[27, 29]
Copper	4.0 × 10^− 2^	N/A	[29]
Lead	3.5 × 10^− 3^	8.5 × 10^− 3^	[27]
Manganese	0.14	N/A	[29]
Nickel	0.02	9.10 × 10^− 1^	[29, 30]

Hazard Quotient which is expressed as a quotient of the Estimated Daily Intake (EDI) to the Reference Dose (RfD) were also of range 6.26 × 10^− 4^ – 106.5.

Values of the THQ and TTHQ (Total Target Hazard Quotient) ranged 0.067–10.34 and 15 respectively.

Cancer risk analysis yielded a range of values of 0.052–19.38. Arsenic and Nickel potentially posed cancer risk to clay consumers (Table 5).

**Table 5:** Calculated Estimated Daily Intake, Hazard Quotient, Target Hazard Quotients and Cancer Risks of the Heavy metals in clay samples

Heavy Metal	Sample	EDI (mg/kg Bw/day)	HQ	THQ	Cancer Risk
Arsenic	Clay	1.88	6.26 × 10^−4^	2.62	2.82
Copper	Clay	2.767	0.069	0.067	N/A
Lead	Clay	0.611	1.75 × 10^− 3^	1.69	0.052
Manganese	Clay	5.44	38.86	0.377	N/A
Nickel	Clay	2.13	106.5	10.34	19.38

*N/A* Not available

Total Target Harzard Quotient (Clay) = 2.62 + 0.067 + 1.69 + 0.377 + 10.34=15

## Discussion

Exposures to chemicals during early life stages can result in adverse effects during the stage when exposure occurred or may not manifest themselves until later stages. Depending on the dose of the chemical and the susceptibility during that life stage to the mode of action of the chemical, effects can range in severity from functional deficits to growth restriction to malformations to ultimate mortality [31, 32].

There has been an increased risks of a range of antagonistic neuro-cognitive developmental effects and increased neonatal and postnatal mortality, spontaneous abortion, suggest: low weight at birth, increased number of still births [32].

Al-Rmalli et al. [9] suggested that an uncertain consumption of 50.0 g of soil taken from an arsenic contaminated area per day is equivalent to 0.370 mg of Arsenic ingestion. From our results, the levels of Arsenic obtained was below the mean exposure level of 3.0 μg/Kg BW/day set by the Joint FAO/WHO Expert Committee on Food Additives [29]. Tayie et al. [10] reported 0.0 (nil) mg/Kg of Arsenic in clay samples in Accra were found to be lower than results obtained in this study. However, Nkansah et al. [27] reported a range of 218–271 ppm from clay samples obtained from different parts of Kumasi Metropolis. Furthermore, Doe et al. [30] also reported Arsenic levels of range 2.7–22.74 μg/g in nine (9) samples of clay samples collected from both Greater Accra and Kumasi of Ghana. Gastric symptoms such as stomach upset, nausea, vomiting, obstruction in the circulatory as well as nervous systems and ultimately death according to Mahurpawah [33], can be a consequence of intakes of large quantities of Arsenic.

Lead concentrations were observed to be below the mean exposure levels and was in contrast with findings of range of values 549–622.92 μg/Kg reported by Nkansah et al. [27]. In line with our results, Tayie et al. [10] reported 2.36 ± 0.08 mg/100 g. Lead consumption can result in adverse health effects such as the dysfunction of some vital organs such as kidneys, liver and heart. Also, multiple organs in the body are targeted by Lead due to systemic toxicity as emphasized by Mahurpawar [33].

Manganese and Nickel levels were also below the set limits. In Tanzania, ranges of 2.3–128 and 2.9–1400 mg/Kg for Nickel and Manganese respectively were reported by Nyanza et al. [34]. Conversely, [30] did not detect any Manganese in the clay samples from Ghana. Essentially, for bone formation and carbohydrate metabolism, Manganese is an essential nutrient involved.

The estimated safe and adequate intake for copper is 1.5–3.0 mg/day. Excessive ingestion of Copper could lead to severe mucosal irritation and corrosion; capillary, hepatic and renal damages; and gastrointestinal and neural disturbances [35]. Copper toxicity seldom occurs but may occur and its consequences may be severe. Intakes of supplements exceeding 3 mg copper/day for a protracted period of time may be a cause for concern [36]. Results obtained in this study was observed to be far below and so contradicted that reported by Nyanza et al. [34] of range 3.9–169 mg/Kg for geophagic soils in Tanzania.

### Health risk assessment

Bonglaisin et al. [37] highlighted that the possibility and the danger of lead or heavy metals getting into human food chain through eating contaminated clay (by birds, animals etc.,) should be dreaded. In children for example, such an exposure would lead to adversarial health consequences on the developing brain, which may result in long-term cerebral deficits as evidenced in records.

Results obtained in this research is in agreement with published findings of [38] in a related study to investigate the health risks posed to pregnant women and children who practice geophagia in Nigeria and found in their study high Arsenic, Lead and Cadmium to be in excess above tolerable limits which yielded hazard quotient and Target Harzard Quotient values of > 1 made clay consumption as pica unsafe and likely to cause adverse effects.

Clay samples obtained from Kumasi, Ghana as worked on by Nkansah et al. [27] recorded Hazard index values of range 0.064. Arsenic was identified as the most hazardous element. A cancer risk assessment confirmed this as a calculated value of 2.2 × 10^− 5^ was reached. Although the hazard index (HI) was below the prescribed unity (1), their cumulative effect was of concern. It could be estimated that the overall non-carcinogenic risk assessment on the health of consumers within the Kumasi Metropolis pointed to more risk via the ingestion route (HI = 0.064).

In another related study by Kamunda et al. [39] in South Africa on toxic elements in soils of a Gold mining basin, they reported HQ values of 2.13 suggestive of non-carcinogenic effects noteworthy to the adult population. Nonetheless, a much greater value of 43.80 was recorded for children oral exposure which posed severe non-carcinogenic risk effects to children living in the mining area. Again, their carcinogenic risks (1.7 × 10^− 4^) have been found to be to be greater than acceptable values.

From China, Liu et al. [40] reported a potential health risk linked with Arsenic and Chromium exposure for residents while risk assessment results which suggested that there were carcinogenic risks of Arsenic and Chromium via corresponding exposure pathways which exceeded the safety limit of 10^− 6^ (the acceptable level of carcinogenic risk for humans). Cancer causing risk of heavy metals is the summation effect of the individual metals contributing to the cancer risk. Results of this study indicated that the sum of cancer risks of the individual metals for the examined toxic metals could pose cancer risk effect to both children and adults through the consumption of examined clay samples contaminated with Lead, Arsenic and Nickel from the Anfoega clay mining site.

## Conclusions

Essential nutrients such as Potassium, Iron, Calcium and Zinc; and toxic metals such as Arsenic, Lead and Mercury were detected in white clay soils mainly from Anfoega, Volta Region and sold for consumption in almost all markets within the region and beyond. The presence of these metals in the clay could be largely due to natural occurrence and a less likely influence of human activities such as handling and or the baking process. The estimated levels of heavy metals contained in 70 g of the geophagic clay consumed by inhabitants in the Ho municipality and beyond were found to be high compared to the Permitted Maximum Tolerable Daily Intake (PMTDI) prescribed by (WHO/FAO). There is a possibility of bioaccumulation after the consumption of these clays by adult pregnant women especially over a long period of time which poses a potential health menace. Human consumption of these clay materials which contain high levels of toxic metals render them obnoxious. Apparently, it is inevitable that geophagy practice will persist despite urbanization and civilization, we therefore support finding ways of reducing heavy metal pollutants in geophagic clays through suitable remediation technology that could minimize the effects of toxic metals on the human system.

Collection of specimen (soils) used in our study complied with guidelines outlined by the Research and Ethics Committee of the University of Health and Allied Sciences, Ho, Ghana.

## Data Availability

The datasets used and/or analysed during the current study available from the corresponding author on reasonable request.

## Abbreviations

EDI: Estimated Daily Intake
HQ: Hazard Quotient
THQ: Target Hazard Quotient
TTHQ: Total Target Hazard Quotient
PMTDI: Permitted Maximum Tolerable Daily Intake
WHO: World Health Organization
FAO: Food and Agriculture Organization
USEPA: United States Environmental Protection Agency
RDI: Recommended Dietary Intake
SPSS: Statistical Package for Social Sciences
